# [4-Bromo-*N*-(pyridin-2-yl­methyl­idene)aniline-κ^2^ *N*,*N*′]iodido(triphenyl­phosphane-κ*P*)copper(I)

**DOI:** 10.1107/S160053681202884X

**Published:** 2012-06-30

**Authors:** Aliakbar Dehno Khalaji, Bahram Bahramian, Khadijeh Jafari, Karla Fejfarová, Michal Dušek

**Affiliations:** a Department of Chemistry, Faculty of Science, Golestan University, Gorgan, Iran; b College of Chemistry, Shahrood University of Technology, Shahrood, Iran; c Institute of Physics ASCR, v.v.i., Na Slovance 2, 182 21 Praha 8, Czech Republic

## Abstract

In the title compound, [CuI(C_12_H_9_BrN_2_)(C_18_H_15_P)], the Cu^I^ ion is bonded to one I atom, one triphenyl­phosphane P atom and two N atoms of the diimine ligand in a distorted tetra­hedral geometry. The Schiff base acts as a chelating ligand and coordinates to the Cu^I^ atom *via* two N atoms. In the diimine ligand, the dihedral angle between the pyridine and bromo­phenyl rings is 19.2 (2)°. In the crystal, mol­ecules are connected by π–π stacking inter­actions between inversion-related pyridine rings [centroid–centroid distance = 3.404 (3) Å].

## Related literature

For related structures and their applications, see: Dehghanpour *et al.* (2006 ▶, 2008 ▶); Saha *et al.* (2010 ▶, 2011*a* ▶,*b* ▶); Habibi *et al.* (2007 ▶); Morshedi *et al.* (2009 ▶); Al-Fayez *et al.* (2007 ▶); Kickelbick *et al.* (2003 ▶); Massa *et al.* (2009 ▶); Chen *et al.* (2012 ▶); Roy *et al.* (2011 ▶). For standard bond lengths, see: Allen *et al.* (1987 ▶).
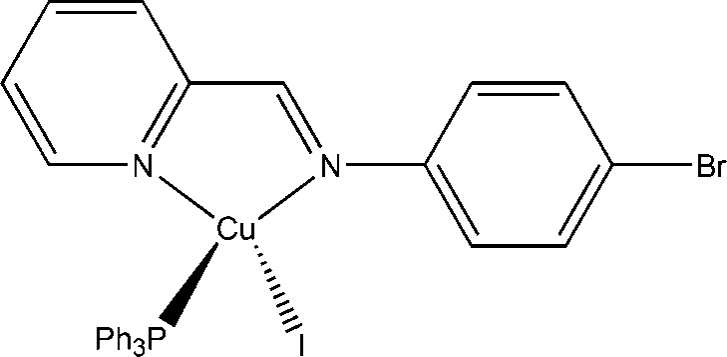


## Experimental

### 

#### Crystal data

[CuI(C_12_H_9_BrN_2_)(C_18_H_15_P)]
*M* *_r_* = 713.9
Monoclinic, 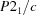

*a* = 10.3141 (5) Å
*b* = 34.7124 (16) Å
*c* = 8.3792 (4) Å
β = 114.321 (6)°
*V* = 2733.7 (3) Å^3^
*Z* = 4
Mo *K*α radiation
μ = 3.47 mm^−1^
*T* = 120 K
0.49 × 0.04 × 0.03 mm

#### Data collection

Agilent Xcalibur diffractometer with an Atlas (Gemini ultra Cu) detector
Absorption correction: multi-scan (*CrysAlis PRO*; Agilent, 2010 ▶) *T*_min_ = 0.914, *T*_max_ = 1.000
14996 measured reflections
5893 independent reflections
4325 reflections with *I* > 3σ(*I*)
*R*_int_ = 0.048

#### Refinement

*R*[*F* ^2^ > 3σ(*F* ^2^)] = 0.038
*wR*(*F* ^2^) = 0.110
*S* = 1.19
5893 reflections
325 parameters
H-atom parameters constrained
Δρ_max_ = 0.70 e Å^−3^
Δρ_min_ = −0.65 e Å^−3^

### 

Data collection: *CrysAlis PRO* (Agilent, 2010 ▶); cell refinement: *CrysAlis PRO*; data reduction: *CrysAlis PRO*; program(s) used to solve structure: *SIR2002* (Burla *et al.*, 2003 ▶); program(s) used to refine structure: *JANA2006* (Petříček *et al.*, 2006 ▶); molecular graphics: *DIAMOND* (Brandenburg & Putz, 2005 ▶); software used to prepare material for publication: *JANA2006*.

## Supplementary Material

Crystal structure: contains datablock(s) global, I. DOI: 10.1107/S160053681202884X/pk2426sup1.cif

Structure factors: contains datablock(s) I. DOI: 10.1107/S160053681202884X/pk2426Isup2.hkl

Additional supplementary materials:  crystallographic information; 3D view; checkCIF report

## Figures and Tables

**Table 1:** Selected bond lengths (Å)

I1—Cu1	2.6386 (7)
Cu1—P1	2.2065 (15)
Cu1—N1	2.119 (5)
Cu1—N2	2.080 (4)

**Fig. 1.**
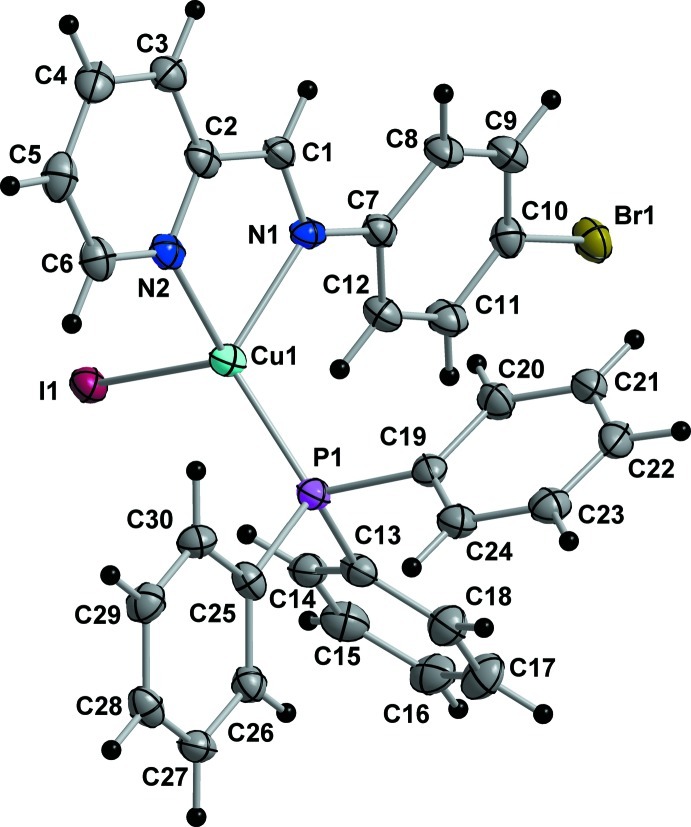
Molecular structure of the title compound. Displacement ellipsoids are drawn at the 50% probability level.

## supplementary crystallographic information

### Comment

The coordination chemistry of copper(I) complexes with bidentate diimine ligands, such as bipyridine and phenanthroline, has received much attention over the last decade due to the many applications of these complexes (Dehghanpour *et al.*, 2006; Saha *et al.*, 2010, 2011*a*, 2011*b*; Habibi *et al.*, 2007). Effort has been devoted to design and synthesis of new Schiff base ligands to control the geometry and properties of copper(I) complexes (Morshedi *et al.*, 2009). Most of the studies have been on tetrahedral copper(I) complexes of the type [Cu(LL)_2_]^+^ and Cu(LL)P_2_]^+^ where LL is a diimine and P is a phosphane (Massa *et al.*, 2009; Dehghanpour *et al.*, 2008; Chen *et al.*, 2012; Roy *et al.*, 2011). Although reports of copper(I) complexes are numerous, limited work has been done on mixed ligand copper(I) complexes of the type [Cu(Schiff base)PX] (*X*= Cl, Br, I) (Dehghanpour *et al.*, 2006; Saha *et al.*, 2010, 2011*a*, 2011*b*; Habibi *et al.*, 2007; Morshedi *et al.*, 2009; Al-Fayez *et al.*, 2007; Kickelbick *et al.*, 2003). This study is a part of our ongoing efforts to synthesize and characterize copper(I) complexes with bidentate Schiff base ligands.

The molecular structure with the atom-numbering scheme is presented in Fig. 1, and the bond lengths (Allen *et al.*, 1987) and angles are generally normal. The copper(I) is coordinated by two nitrogen atoms of the bidentate Schiff-base ligand, one P atom of triphenylphosphane and one I atom. Although a tetrahedral geometry might be expected for a four coordinate copper(I) centre, the geometry around the copper(I) ion is distorted by the restricting bite angle N1—Cu1—N2 [79.3 (2)°] of the chelating Schiff-base ligand.

### Experimental

To a stirring solution of 190 mg (1 mmol) CuI in 5 ml of acetonitrile was added dropwise 263 mg (1 mmol) of triphenylphosphane in 5 ml acetonitrile. The mixture was stirred for 30 min and then 261 mg (1 mmol) of ligand, 4-bromophenylpyridine-2-ylmethyleneamine, in 10 ml acetonitrile was added and stirred for an additional 20 min. The volume of the solvent was reduced under vacuum to about 5 ml. The diffusion of diethyl ether vapor into the concentration solution gave dark red crystals. The crystals were filtered off and washed with Et_2_O. Yield: 65%. *Anal*. Calc. for C_30_H_24_N_2_CuPBrI: C, 50.48; H, 3.38; N, 3.93%. Found: C, 50.55; H, 3.51; N, 3.78%.

### Refinement

All hydrogen atoms were positioned geometrically and treated as riding on their parent atoms. The isotropic atomic displacement parameters of hydrogen atoms were evaluated as 1.2×*U*_eq_ of the parent atom.

### Crystal data

**Table:**

[CuI(C_12_H_9_BrN_2_)(C_18_H_15_P)]	*F*(000) = 1400
*M**_r_* = 713.9	*D*_x_ = 1.734 Mg m^−^^3^
Monoclinic, *P*2_1_/*c*	Mo *K*α radiation, λ = 0.7107 Å
Hall symbol: -P 2ybc	Cell parameters from 5306 reflections
*a* = 10.3141 (5) Å	θ = 2.9–27.0°
*b* = 34.7124 (16) Å	µ = 3.47 mm^−^^1^
*c* = 8.3792 (4) Å	*T* = 120 K
β = 114.321 (6)°	Needle, red
*V* = 2733.7 (3) Å^3^	0.49 × 0.04 × 0.03 mm
*Z* = 4	

### Data collection

**Table:**

Agilent Xcalibur diffractometer with an Atlas (Gemini ultra Cu) detector	5893 independent reflections
Radiation source: Enhance (Mo) X-ray Source	4325 reflections with *I* > 3σ(*I*)
Graphite monochromator	*R*_int_ = 0.048
Detector resolution: 10.4 pixels mm^-1^	θ_max_ = 27.2°, θ_min_ = 2.9°
Rotation method data acquisition using ω scans	*h* = −12→13
Absorption correction: multi-scan (*CrysAlis PRO*; Agilent, 2010)	*k* = −44→43
*T*_min_ = 0.914, *T*_max_ = 1.000	*l* = −10→10
14996 measured reflections	

### Refinement

**Table:**

Refinement on *F*^2^	96 constraints
*R*[*F*^2^ > 2σ(*F*^2^)] = 0.038	H-atom parameters constrained
*wR*(*F*^2^) = 0.110	Weighting scheme based on measured s.u.'s *w* = 1/(σ^2^(*I*) + 0.0016*I*^2^)
*S* = 1.19	(Δ/σ)_max_ = 0.028
5893 reflections	Δρ_max_ = 0.70 e Å^−^^3^
325 parameters	Δρ_min_ = −0.65 e Å^−^^3^
0 restraints	

### Special details

**Table:**

Experimental. CrysAlisPro (Agilent, 2010) Empirical absorption correction using spherical harmonics, implemented in SCALE3 ABSPACK scaling algorithm.
Refinement. The refinement was carried out against all reflections. The conventional *R*-factor is always based on *F*. The goodness of fit as well as the weighted *R*-factor are based on *F* and *F*^2^ for refinement carried out on *F* and *F*^2^, respectively. The threshold expression is used only for calculating *R*-factors *etc*. and it is not relevant to the choice of reflections for refinement.The program used for refinement, Jana2006, uses the weighting scheme based on the experimental expectations, see _refine_ls_weighting_details, that does not force *S* to be one. Therefore the values of *S* are usually larger than the ones from the *SHELX* program.

### Fractional atomic coordinates and isotropic or equivalent isotropic displacement parameters (Å^2^)

**Table:**

	*x*	*y*	*z*	*U*_iso_*/*U*_eq_	
I1	0.78044 (3)	0.068800 (9)	0.72718 (4)	0.02639 (13)	
Cu1	0.59846 (6)	0.079263 (17)	0.39909 (7)	0.0238 (2)	
Br1	1.00314 (6)	0.252746 (15)	0.34867 (7)	0.0330 (2)	
P1	0.41099 (13)	0.11517 (4)	0.35881 (16)	0.0221 (4)	
N1	0.7265 (4)	0.09533 (11)	0.2668 (5)	0.0210 (14)	
N2	0.6081 (4)	0.02740 (12)	0.2802 (5)	0.0239 (15)	
C1	0.7562 (5)	0.06704 (13)	0.1899 (6)	0.0246 (18)	
C2	0.6948 (5)	0.02930 (14)	0.1929 (6)	0.0249 (17)	
C3	0.7260 (5)	−0.00216 (14)	0.1148 (6)	0.0277 (18)	
C4	0.6666 (5)	−0.03762 (15)	0.1227 (6)	0.032 (2)	
C5	0.5758 (5)	−0.03981 (15)	0.2079 (6)	0.0315 (19)	
C6	0.5512 (5)	−0.00660 (14)	0.2856 (6)	0.0275 (18)	
C7	0.7877 (5)	0.13217 (14)	0.2721 (6)	0.0229 (17)	
C8	0.8508 (5)	0.14430 (14)	0.1607 (6)	0.029 (2)	
C9	0.9133 (5)	0.18024 (15)	0.1828 (6)	0.030 (2)	
C10	0.9110 (5)	0.20401 (14)	0.3131 (6)	0.0259 (18)	
C11	0.8463 (5)	0.19317 (14)	0.4213 (6)	0.0292 (19)	
C12	0.7854 (5)	0.15713 (14)	0.4002 (6)	0.0257 (18)	
C13	0.4456 (5)	0.16036 (14)	0.4825 (6)	0.0247 (18)	
C14	0.5628 (5)	0.16325 (14)	0.6392 (6)	0.0270 (19)	
C15	0.5972 (5)	0.19795 (15)	0.7293 (7)	0.033 (2)	
C16	0.5145 (6)	0.23034 (15)	0.6643 (7)	0.035 (2)	
C17	0.3958 (6)	0.22751 (15)	0.5099 (7)	0.037 (2)	
C18	0.3608 (6)	0.19342 (14)	0.4189 (7)	0.033 (2)	
C19	0.3142 (5)	0.13315 (13)	0.1366 (6)	0.0246 (18)	
C20	0.3927 (5)	0.15205 (14)	0.0564 (6)	0.0274 (19)	
C21	0.3251 (5)	0.16944 (14)	−0.1052 (6)	0.029 (2)	
C22	0.1783 (5)	0.16791 (14)	−0.1889 (6)	0.031 (2)	
C23	0.1010 (5)	0.14853 (14)	−0.1132 (6)	0.0285 (19)	
C24	0.1677 (5)	0.13116 (14)	0.0487 (6)	0.0271 (19)	
C25	0.2723 (5)	0.09130 (14)	0.4047 (6)	0.0235 (17)	
C26	0.2011 (5)	0.10822 (14)	0.4975 (6)	0.0248 (18)	
C27	0.0987 (5)	0.08829 (14)	0.5314 (6)	0.0255 (18)	
C28	0.0620 (5)	0.05149 (14)	0.4687 (6)	0.0265 (18)	
C29	0.1315 (5)	0.03393 (15)	0.3733 (6)	0.030 (2)	
C30	0.2352 (5)	0.05376 (14)	0.3424 (6)	0.0263 (19)	
H1	0.81789	0.070579	0.131067	0.0295*	
H3	0.787927	0.000399	0.055749	0.0332*	
H4	0.687643	−0.060078	0.070836	0.0387*	
H5	0.53086	−0.063714	0.212969	0.0378*	
H6	0.490079	−0.008491	0.346082	0.033*	
H8	0.85054	0.127607	0.069147	0.0346*	
H9	0.95784	0.188549	0.108178	0.0365*	
H11	0.843766	0.210414	0.509557	0.0351*	
H12	0.740691	0.149188	0.475133	0.0308*	
H14	0.621452	0.141025	0.686939	0.0324*	
H15	0.679671	0.199445	0.838399	0.0399*	
H16	0.539722	0.254385	0.726113	0.0422*	
H17	0.33634	0.249691	0.46463	0.0447*	
H18	0.277444	0.192115	0.310656	0.0391*	
H20	0.494659	0.152842	0.114923	0.0329*	
H21	0.379189	0.182424	−0.158621	0.0346*	
H22	0.129947	0.180362	−0.300197	0.0375*	
H23	−0.000701	0.147118	−0.173989	0.0341*	
H24	0.112699	0.117791	0.099932	0.0325*	
H26	0.223603	0.134233	0.538479	0.0297*	
H27	0.05353	0.100187	0.598676	0.0306*	
H28	−0.010308	0.037787	0.48966	0.0318*	
H29	0.106618	0.008169	0.329775	0.0359*	
H30	0.282097	0.041643	0.277593	0.0315*	

### Atomic displacement parameters (Å^2^)

**Table:**

	*U*^11^	*U*^22^	*U*^33^	*U*^12^	*U*^13^	*U*^23^
I1	0.03169 (19)	0.02779 (19)	0.02021 (17)	0.00070 (14)	0.01123 (14)	−0.00064 (12)
Cu1	0.0255 (3)	0.0265 (3)	0.0224 (3)	0.0003 (3)	0.0128 (3)	−0.0009 (2)
Br1	0.0418 (3)	0.0281 (3)	0.0311 (3)	−0.0068 (2)	0.0171 (3)	−0.0001 (2)
P1	0.0252 (6)	0.0227 (6)	0.0213 (6)	−0.0001 (5)	0.0125 (5)	−0.0004 (5)
N1	0.022 (2)	0.025 (2)	0.0164 (19)	0.0010 (17)	0.0088 (17)	−0.0026 (15)
N2	0.025 (2)	0.028 (2)	0.0180 (19)	0.0004 (18)	0.0078 (17)	0.0006 (16)
C1	0.024 (2)	0.032 (3)	0.020 (2)	0.006 (2)	0.012 (2)	0.0057 (19)
C2	0.024 (2)	0.032 (3)	0.015 (2)	0.001 (2)	0.004 (2)	0.0013 (19)
C3	0.031 (3)	0.030 (3)	0.022 (2)	0.003 (2)	0.010 (2)	−0.001 (2)
C4	0.045 (3)	0.028 (3)	0.020 (2)	0.005 (2)	0.010 (2)	−0.004 (2)
C5	0.033 (3)	0.026 (3)	0.027 (3)	−0.002 (2)	0.004 (2)	0.001 (2)
C6	0.027 (3)	0.029 (3)	0.021 (2)	−0.003 (2)	0.005 (2)	0.0006 (19)
C7	0.022 (2)	0.028 (3)	0.019 (2)	0.001 (2)	0.009 (2)	0.0023 (19)
C8	0.040 (3)	0.029 (3)	0.022 (3)	0.001 (2)	0.018 (2)	−0.002 (2)
C9	0.035 (3)	0.035 (3)	0.027 (3)	0.000 (2)	0.019 (2)	0.002 (2)
C10	0.028 (3)	0.026 (3)	0.022 (2)	−0.001 (2)	0.008 (2)	0.0009 (19)
C11	0.037 (3)	0.030 (3)	0.023 (2)	−0.002 (2)	0.016 (2)	−0.005 (2)
C12	0.029 (3)	0.030 (3)	0.022 (2)	−0.002 (2)	0.013 (2)	−0.0013 (19)
C13	0.029 (3)	0.026 (3)	0.024 (2)	−0.003 (2)	0.016 (2)	−0.0022 (19)
C14	0.030 (3)	0.028 (3)	0.028 (3)	0.001 (2)	0.017 (2)	−0.002 (2)
C15	0.031 (3)	0.039 (3)	0.031 (3)	−0.005 (2)	0.013 (2)	−0.007 (2)
C16	0.043 (3)	0.023 (3)	0.045 (3)	−0.003 (2)	0.024 (3)	−0.008 (2)
C17	0.043 (3)	0.022 (3)	0.047 (3)	0.000 (2)	0.018 (3)	−0.004 (2)
C18	0.036 (3)	0.028 (3)	0.034 (3)	−0.001 (2)	0.016 (3)	−0.001 (2)
C19	0.035 (3)	0.019 (2)	0.024 (2)	0.002 (2)	0.015 (2)	−0.0018 (18)
C20	0.027 (3)	0.028 (3)	0.030 (3)	−0.002 (2)	0.014 (2)	0.004 (2)
C21	0.038 (3)	0.026 (3)	0.030 (3)	0.004 (2)	0.021 (2)	0.003 (2)
C22	0.043 (3)	0.031 (3)	0.020 (2)	0.010 (2)	0.012 (2)	0.002 (2)
C23	0.029 (3)	0.031 (3)	0.025 (3)	0.001 (2)	0.010 (2)	−0.004 (2)
C24	0.032 (3)	0.028 (3)	0.025 (3)	0.001 (2)	0.015 (2)	−0.002 (2)
C25	0.025 (2)	0.027 (3)	0.019 (2)	−0.002 (2)	0.009 (2)	0.0028 (18)
C26	0.029 (3)	0.024 (3)	0.022 (2)	0.000 (2)	0.011 (2)	0.0003 (18)
C27	0.025 (2)	0.031 (3)	0.024 (2)	0.002 (2)	0.013 (2)	−0.001 (2)
C28	0.023 (2)	0.031 (3)	0.025 (2)	−0.005 (2)	0.009 (2)	0.003 (2)
C29	0.035 (3)	0.023 (3)	0.032 (3)	0.001 (2)	0.013 (2)	−0.003 (2)
C30	0.029 (3)	0.026 (3)	0.026 (3)	0.002 (2)	0.014 (2)	−0.003 (2)

### Geometric parameters (Å, º)

**Table:**

I1—Cu1	2.6386 (7)	C13—C18	1.408 (7)
Cu1—P1	2.2065 (15)	C14—C15	1.388 (7)
Cu1—N1	2.119 (5)	C14—H14	0.96
Cu1—N2	2.080 (4)	C15—C16	1.380 (7)
Br1—C10	1.903 (5)	C15—H15	0.96
P1—C13	1.832 (5)	C16—C17	1.370 (7)
P1—C19	1.823 (4)	C16—H16	0.96
P1—C25	1.826 (6)	C17—C18	1.373 (7)
N1—C1	1.279 (7)	C17—H17	0.96
N1—C7	1.419 (6)	C18—H18	0.96
N2—C2	1.371 (7)	C19—C20	1.409 (8)
N2—C6	1.327 (6)	C19—C24	1.384 (7)
C1—C2	1.460 (7)	C20—C21	1.381 (6)
C1—H1	0.96	C20—H20	0.96
C2—C3	1.377 (7)	C21—C22	1.383 (7)
C3—C4	1.388 (7)	C21—H21	0.96
C3—H3	0.96	C22—C23	1.382 (9)
C4—C5	1.394 (9)	C22—H22	0.96
C4—H4	0.96	C23—C24	1.381 (6)
C5—C6	1.398 (8)	C23—H23	0.96
C5—H5	0.96	C24—H24	0.96
C6—H6	0.96	C25—C26	1.399 (8)
C7—C8	1.405 (8)	C25—C30	1.397 (7)
C7—C12	1.387 (7)	C26—C27	1.386 (8)
C8—C9	1.381 (7)	C26—H26	0.96
C8—H8	0.96	C27—C28	1.374 (7)
C9—C10	1.377 (8)	C27—H27	0.96
C9—H9	0.96	C28—C29	1.413 (8)
C10—C11	1.381 (8)	C28—H28	0.96
C11—C12	1.378 (7)	C29—C30	1.384 (8)
C11—H11	0.96	C29—H29	0.96
C12—H12	0.96	C30—H30	0.96
C13—C14	1.374 (6)		
			
I1—Cu1—P1	116.08 (4)	P1—C13—C14	119.5 (4)
I1—Cu1—N1	104.61 (8)	P1—C13—C18	122.6 (3)
I1—Cu1—N2	103.06 (9)	C14—C13—C18	117.8 (4)
P1—Cu1—N1	117.84 (11)	C13—C14—C15	120.7 (4)
P1—Cu1—N2	128.86 (10)	C13—C14—H14	119.66
N1—Cu1—N2	79.31 (17)	C15—C14—H14	119.6609
Cu1—P1—C13	116.28 (16)	C14—C15—C16	121.0 (4)
Cu1—P1—C19	115.2 (2)	C14—C15—H15	119.5162
Cu1—P1—C25	115.14 (16)	C16—C15—H15	119.5164
C13—P1—C19	100.5 (2)	C15—C16—C17	118.7 (5)
C13—P1—C25	104.8 (3)	C15—C16—H16	120.6387
C19—P1—C25	102.9 (2)	C17—C16—H16	120.6398
Cu1—N1—C1	113.1 (3)	C16—C17—C18	121.0 (5)
Cu1—N1—C7	125.9 (3)	C16—C17—H17	119.5157
C1—N1—C7	120.8 (5)	C18—C17—H17	119.5157
Cu1—N2—C2	112.6 (3)	C13—C18—C17	120.8 (4)
Cu1—N2—C6	130.1 (4)	C13—C18—H18	119.5977
C2—N2—C6	117.1 (4)	C17—C18—H18	119.598
N1—C1—C2	119.0 (5)	P1—C19—C20	117.8 (3)
N1—C1—H1	120.4965	P1—C19—C24	123.0 (4)
C2—C1—H1	120.4972	C20—C19—C24	119.1 (4)
N2—C2—C1	115.8 (5)	C19—C20—C21	120.9 (5)
N2—C2—C3	123.1 (5)	C19—C20—H20	119.5623
C1—C2—C3	121.1 (5)	C21—C20—H20	119.5601
C2—C3—C4	119.1 (6)	C20—C21—C22	119.1 (5)
C2—C3—H3	120.4409	C20—C21—H21	120.4332
C4—C3—H3	120.442	C22—C21—H21	120.4324
C3—C4—C5	118.4 (5)	C21—C22—C23	120.3 (4)
C3—C4—H4	120.8107	C21—C22—H22	119.8737
C5—C4—H4	120.8119	C23—C22—H22	119.8726
C4—C5—C6	118.9 (5)	C22—C23—C24	121.0 (5)
C4—C5—H5	120.5658	C22—C23—H23	119.4855
C6—C5—H5	120.566	C24—C23—H23	119.4861
N2—C6—C5	123.4 (6)	C19—C24—C23	119.6 (5)
N2—C6—H6	118.315	C19—C24—H24	120.2034
C5—C6—H6	118.317	C23—C24—H24	120.2026
N1—C7—C8	124.8 (4)	P1—C25—C26	124.2 (4)
N1—C7—C12	116.0 (5)	P1—C25—C30	117.5 (4)
C8—C7—C12	119.2 (5)	C26—C25—C30	118.3 (5)
C7—C8—C9	120.0 (5)	C25—C26—C27	121.5 (4)
C7—C8—H8	120.0129	C25—C26—H26	119.2398
C9—C8—H8	120.0148	C27—C26—H26	119.2411
C8—C9—C10	119.2 (6)	C26—C27—C28	119.9 (5)
C8—C9—H9	120.3841	C26—C27—H27	120.0454
C10—C9—H9	120.3841	C28—C27—H27	120.0454
Br1—C10—C9	119.1 (4)	C27—C28—C29	119.6 (5)
Br1—C10—C11	119.0 (4)	C27—C28—H28	120.2082
C9—C10—C11	121.9 (5)	C29—C28—H28	120.2108
C10—C11—C12	118.7 (5)	C28—C29—C30	120.2 (5)
C10—C11—H11	120.6268	C28—C29—H29	119.904
C12—C11—H11	120.6295	C30—C29—H29	119.9041
C7—C12—C11	120.9 (5)	C25—C30—C29	120.5 (5)
C7—C12—H12	119.5252	C25—C30—H30	119.7526
C11—C12—H12	119.5264	C29—C30—H30	119.7517
